# Supplementary material for: Causal Relationship between Aging and Anorexia Nervosa: A White-Matter-Microstructure-Mediated Mendelian Randomization Analysis
Source: Biomedicines. 2024 Aug 16;12(8):1874. doi: 10.3390/biomedicines12081874 (PMC11351342; doi:10.3390/biomedicines12081874)
Supplement: Supplementary file 1 [file biomedicines-12-01874-s001.zip › Supplementary Figure/Figure S7/Single SNP plot of MR analysis for ubm-b-1473 to AN.pdf]

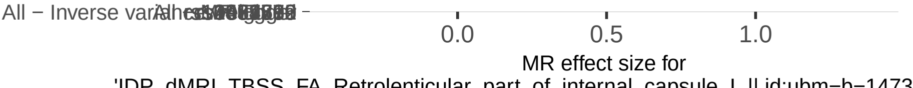
All − Inverse variance weighted
0.0
0.5
1.0
MR effect size for
'IDP_dMRI_TBSS_FA_Retrolenticular_part_of_internal_capsule_L || id:ubm−b−1473
